# Le cavernome porte chez l'enfant: a propos de 11 cas

**DOI:** 10.11604/pamj.2014.19.277.5571

**Published:** 2014-11-14

**Authors:** Tadmori Ilham, Lakhdar Idrissi Mounia, Hida Moustapha

**Affiliations:** 1 Service Pédiatrie, Département Mère-Enfant, CHU Hassan II, Fès, Maroc

**Keywords:** Obstruction chronique, enfant, hémorragie digestive, chronic obstruction, child, gastrointestinal bleeding

## Abstract

Le cavernome portal est la conséquence d'une occlusion chronique, du système porte extra-hépatique formé d'un réseau de veines dont le calibre est augmenté et au sein desquelles chemine un sang portal hépatopéte. Chez l'enfant, est une cause majeure d'hypertension portale dite «pré ou infra-hépatique» ou encore «extra-hépatique». Onze cas de cavernome porte parmi 78 cas d'hypertension portale ont été colligés au service sur une période allant du Janvier 2003 au Septembre 2012. L’âge de nos patients variait entre 2 et 15 ans et le sexe ratio est de 1,75. Tous nos patients étaient admis au stade d'hypertension portale avec la splénomégalie SMG (100% des cas); hémorragies digestives (63%); ascite (36%); la circulation veineuse collatérale CVC et l'hépatomégalie HMG (27%). L'exploration endoscopique a objectivé la présence de varices ‘sophagiennes dans tous les cas avec une gastropathie hypertensive dans 27% et des varices ectopiques chez 36%. Les perturbations biologiques étaient dominées par la pancytopénie. Le bilan de thrombophilie était demandé pour tous les malades mais réalisé mais n'est réalisé que chez trois revenus normaux chez deux et a objectivé une baisse de protéine S chez le troisième. L’échographie abdominale était le moyen de diagnostic positif et l’écho-doppler a confirmé l'HTP chez tous nos patients. Aucun de nos malades n'a pu être opéré pour le moment. La ligature a été réalisée chez 54,5%. Dix patients ont nécessité une transfusion sanguine. L’évolution globale de nos patients est favorable. La durée moyenne d’évolution du cavernome porte chez nos patients est de quatre ans.

## Introduction

Un cavernome portal est un réseau formé de veines dont le calibre, initialement millimétrique ou microscopique, au sein duquel chemine un sang portal hépatopéte. Il est la conséquence d'une occlusion chronique du système porte extra-hépatique. Ces veines dilatées deviennent tortueuses et réalisent un véritable réseau d'allure angiomatose, mais qui reste pourtant insuffisant pour drainer l'ensemble de la circulation splanchnique, d'où l'installation d'une hypertension portale. Chez l'enfant, le cavernome portal est une cause majeure d'hypertension portale dite «pré ou infra-hépatique» ou encore «extra-hépatique», dont le diagnostic repose principalement sur l'imagerie.

## Méthodes

Notre travail est une étude rétrospective et prospective colligeant 11 cas de cavernome porte parmi 78 cas d'hypertension portale au sein du service de pédiatrie au CHU Hassan II de Fès sur une période allant du Janvier 2003 au septembre 2012. Le but de cette étude est de rapporter l'expérience de notre service.

## Résultats

L′âge de nos patients est compris entre 24mois et 15ans, avec une moyenne d′âge de 8 ans et dont 45,5% des patients sont âgés entre 8ans et 12 ans. Dans notre série on note une prédominance masculine avec un sex-ratio de 1,7. Les antecedents (ATCD) chez nos patients on trouve la consanguinité parentale dans deux cas; la prise médicamenteuse (l'AINS et l'aspirine) était rapportée dans 18% des cas provocant l'hémorragie digestive. Un de nos patients avait une infection abdominale au cours de la découverte du cavernome porte. Aucun de nos patients n'avait un ATCD d'hospitalisation néonatale ni de cathétérisme ombilical; ni de chirurgie antérieure ni de traumatisme abdominal. Les ATCD thromboemboliques ou de thrombophilie dans la famille n'ont été recherchés en aucun cas. Le motif d'hospitalisation était dominé par l'hémorragie digestive dans 91% des cas et pour la distension abdominale était isolée dans un cas et associée à une hémorragie digestive dans deux cas. Tous nos patients étaient admis en stade d'HTP et dont l'examen physique a objectivé une splénomégalie dans 100%; une hémorragie digestive dans 64%; une distension abdominale et une pâleur dans 55%; une ascite a été retrouvée dans 37%; une hépatomégalie et une circulation veineuse collatérale ont été objectivées dans 27% et la douleur abdominale type pesanteur dans 18% des cas.

**Figure 1:**
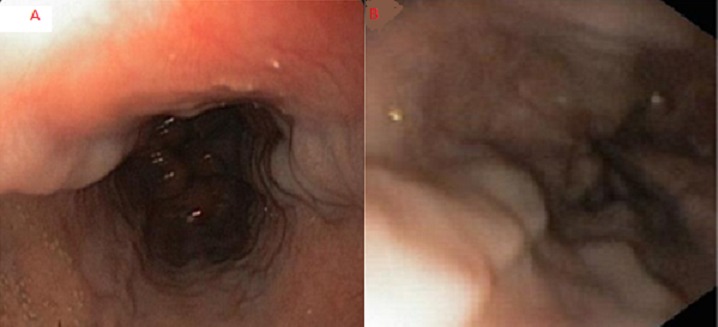
(A) varices œsophagiennes Stade I et (B) Stade II

**Figure 2:**
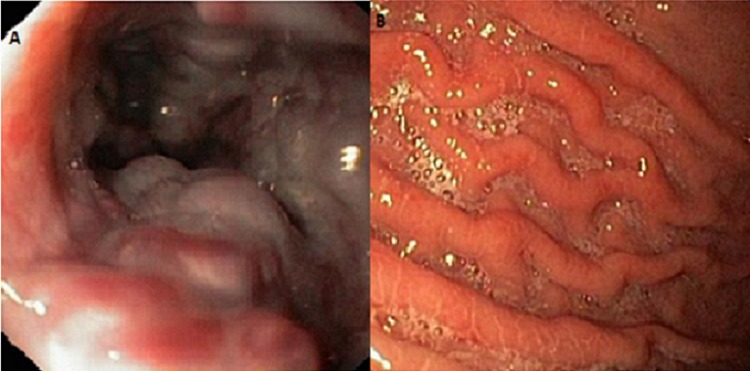
(A) varices œsophagiennes Stade III et (B) image endoscopique d'une gastropathie hypertensive

**Figure 3:**
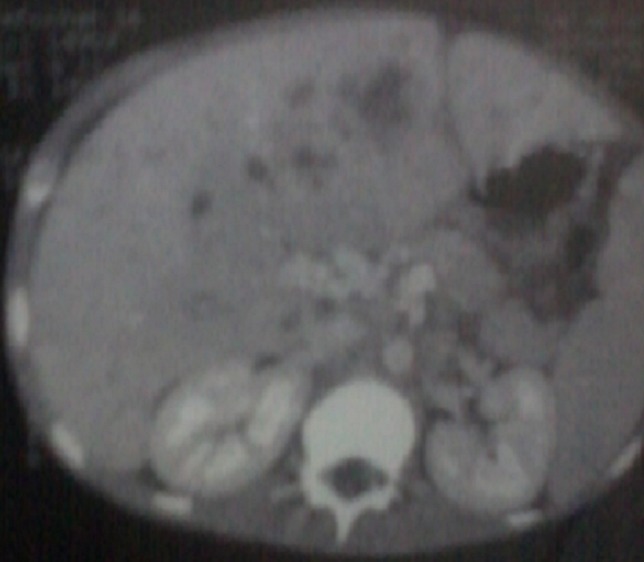
Coupe tomodensitométrie après injection de produit de contraste mettant en évidence de nombreuses structures vasculaires serpigineuses correspondant à un cavernome

**Figure 4:**
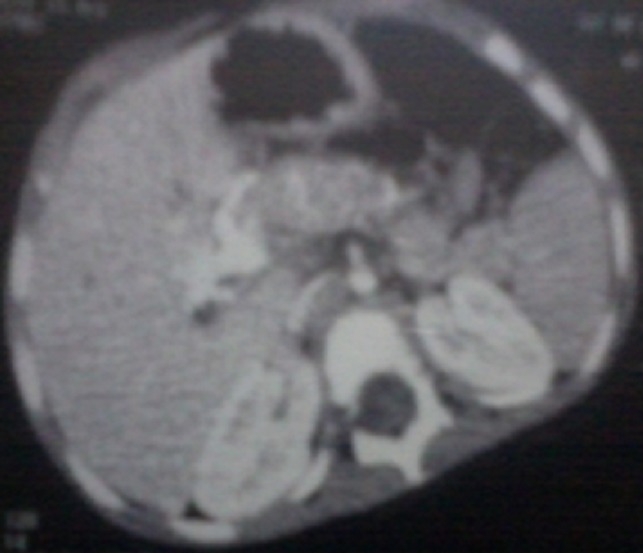
Coupe tomodensitométrie après injection de produit de contraste mettant en évidence de nombreuses structures vasculaires (flèche) du hile hépatique correspondant à un cavernome

**Figure 5:**
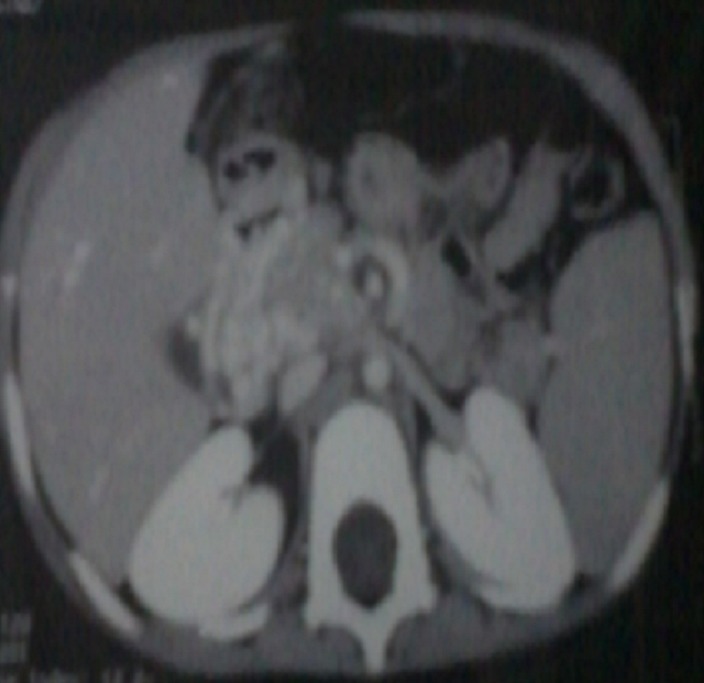
Coupe tomodensitométrie après injection de produit de contraste mettant en évidence de nombreuses structures vasculaires serpigineuses au niveau du hile hépatique correspondant à un cavernome

Pour les examens biologiques la NFS ont affirmé, dans 100% des cas, l'existence d'un hypersplénisme, avec une pancytopénie dans quatre cas soit 36% et une bi-cytopénie dans 64% des cas. Le bilan d'hémostase, réalisé chez tous nos malades, est revenu perturbé chez 64%. Un bilan hépatique et lipidique a été réalisé et revenu normal. L’électrophorèse des protéines plasmatiques réalisée pour six cas et a objectivé une hypo-protidémie avec hypo-albuminémie dans quatre cas. La sérologie hépatique B et C est réalisée chez cinq patients et qui est revenue négative. Le bilan de thrombophilie a été réalisé dans trois cas; il est revenu normal chez deux malades et a objectivé un dosage de protéine S bas chez le troisième. L'exploration endoscopique, était réalisé chez tous nos patients, a objectivé la présence de varices œsophagiennes (VO) dans 100% des cas, stade I chez deux patients, stade II chez trois patients, stades variables I, II et III chez six patients. En plus des VO, des varices ectopiques étaient présents chez quatre cas. Elles sont cardiotubérositaires chez trois cas et duodénales chez un seul. Une gastropathie hypertensive dans 27% des cas (Figure 1, Figure 2). L’écho-doppler a été réalisée chez tous les malades. Elle était le moyen de diagnostic positif et a confirmé l'HTP sur cavernome porte chez tous nos patients à travers des signes directs d'une vascularisation porto-portale ou des signes indirects à savoir; une paroi vésiculaire épaissie et un petit épiploon épaissi. L'exploration hépatobiliaire a objectivé un foie d’échogénicité homogène dans 73% cas et hétérogène dans 27% cas. L'angioscanner a été fait chez trois patients dans un but préopératoire pour mieux explorer la vascularisation portale et ses différentes anastomoses. L'angio-scanner n'a pas montré de réseau porte extra-hépatique avec absence d'individualisation de la veine splénique en sa totalité (visible que dans sa partie proximale) dans un cas et un shunt spléno-rénal gauche dans deux cas (Figure 3, Figure 4, Figure 5).

Le traitement chez nos patients est basé sur le traitement endoscopique par la ligature élastique des varices a constitué le seul mode thérapeutique dans notre série et la prophylaxie des hémorragies dues à l'hypertension portale par β-bloquants. Les six enfants qui ont bénéficié de la ligature, cinq d'entre eux ont eu des VO stade III, et un seul patient avait des varices cardiotubérositaires. La ligature des VO a été réalisée en urgence initialement chez trois cas vu les hémorragies digestives de grande abondance et ce après stabilisation de l’état hémodynamique. Pour les trois autres patients la ligature a été programmée. Le nombre total des séances de ligature était 17 séances avec une moyenne de 2 à 3 séances par patient. Elle a permis l’éradication des VO dans quatre cas en association aux β-bloquants. Deux de nos patients continuent toujours à faire des récidives hémorragiques et bénéficient de séances de ligatures malgré le traitement par les β-bloquants. La ligature n’était pas réalisée chez cinq cas, vu la stabilité grâce aux β-bloquants chez quatre patients; et elle n’était pas faite pour d'emblé pour une patiente qui avait des varices duodénales en raison de la difficulté d'accessibilité endoscopique. La transfusion sanguine a été nécessaire chez dix patients. Le traitement par les β-bloquants (Propranolol à dose de 2mg/kg/j) a été prescrit et pris chez tous nos malades. Chez quatre patients; les β-bloquants en association avec la ligature ont permis l’éradication des VO. Les autres patients sont restés stables grâce au β-bloquant seul. Aucun de nos malades n'a pu être opéré pour le moment alors que trois étaient candidats à la chirurgie. L’évolution globale, de nos patients, sous traitement est favorable chez huit cas. Un patient n'a pas présenté de saignement depuis le diagnostic et trois cas nécessitent toujours des transfusions vu les récidives hémorragiques. La durée moyenne d’évolution chez les patients porteurs d'un cavernome porte est 3,5ans. L’évolution des patients n'ayant pas bénéficié de la ligature des VO était favorable chez quatre cas alors que le cinquième présente toujours des récidives hémorragiques vu les varices ectopiques. Dans notre série aucun décès n'a été noté.

## Discussion

Le cavernome portal est la conséquence d'une occlusion chronique du système porte extra-hépatique. Il est formé d'un réseau de veines dont le calibre, initialement millimétrique ou microscopique, est augmenté et au sein desquelles chemine un sang portal hépatopète. Chez l'enfant, le cavernome portal est une cause majeure d'hypertension portale dite «pré ou infra-hépatique» ou encore «extra-hépatique» [1]. Son incidence est variable d'une série à l'autre. Dans notre étude, on a colligé 11 cas en 10ans, soit une incidence de presque un cas par an, ce qui reste un chiffre faible en comparaison avec les autres séries [1]. Le cavernome portal survient à tout âge avec une moyenne allant de 10 à 30 ans selon les différentes séries [2, 3] L’âge dans notre série ne diffère pas de ceux enregistrés dans la littérature, avec une médiane de 8ans et des extrêmes de 2ans et 15ans à la découverte de la maladie. Le cavernome portal ne semble pas une pathologie liée au sexe. D'une part, des études [2, 4, 5], ont noté une prédominance masculine avec, respectivement, un sex-ratio de 2,3/1,2/ et 2. D'autre part, les autres séries [1, 3] ont noté une prédominance du sexe féminin avec, respectivement, un sex-ratio de 2 et 1,2. Dans notre série, on note une prédominance masculine avec un sex-ratio de 1,75. Aucune malformation n'a été observée chez nos patients, ce qui concorde avec leur rareté dans la littérature. Un ATCD de cathétérisme ombilical en vue d'une réanimation néo-natale expose à la thrombose portale. Aucun de nos patients n'avait subi ce geste à sa période néo-natale. Le cathétérisme ombilical pour la réanimation d'un prématuré a été enregistré dans une série [1]. Un ATCD de traumatisme abdominal, qu'il soit accidentel ou chirurgical, pourrait être responsable d'une thrombose abdominal. De même un ATCD de thrombose veineuse à répétition ou survenue à un âge jeune dans la famille pourrait mener au diagnostic d'un état thrombotique ou de thrombophilie familiale. On n'en a noté aucun de ces ATCD dans notre série. Un ATCD d'infection abdominale quelle que soit sa localisation pourrait être responsable d'une thrombose abdominal. Un ATCD d'infection hépatique à type de micro-abcès a été noté chez un de nos patients.

Les hémorragies, le plus souvent digestives, représentent le motif de consultation le plus fréquent et dont le pourcentage est variable selon les séries 80%, 77% et 90% des cas [1–3]. Il s'agit surtout d'hématémèse suivis d’épistaxis. La splénomégalie constitue le deuxième motif de consultation chez les enfants. Elle révèle la maladie dans 22% [2] et 9% [1] des cas selon les séries. Dans notre étude, les hémorragies sont révélatrices dans 91% et la SMG dans 36,5% des cas. Les signes cliniques sont représentés par les Hémorragies digestives à type d'hématémèses Ces hémorragies digetives hautes sont lies soit à la rupture de varices (œsophagiennes, cardiotubérositaires) ou au par saignement d'une gastropathie hypertensive, soit spontanément ou à la suite d'une prise médicamenteuse (AINS ou aspirine) Elles sont favorisées favorisé par des troubles de la coagulation souvent présents à cause de la thrombose et de l'hypersplénisme [6]. Ces hématémèses qui sont représentées par des hématémèses qui sont souvent suivies de mælena ou des réctorragies par rupture des varices ectopiques en particulier au niveau du colon et de rectum (hémorroïdes). On note que les hémorroides n'ont été recherchées en aucun cas dans notre série et rarement rapportées dans la littérature (2%) [6]. On note également une fréquence des hémorragies extradigestives faites d’épistaxis, de gingivorragies, de lésions purpuriques... expliquées par la thrombopénie et les troubles existants de la coagulation. Dans notre série, on a enregistré des épistaxis dans un cas. La fréquence des hémorragies dans notre série est de 63%, faites d'hémateméses dans 54,5% des cas, ce qui rejoint les données de la littérature: 80% [2], 77% [3], 46% [7], 40% [8] et 90% [1].

La SMG est un signe commun que présentent les patients avec une HTP soit extra ou intra hépatique. Elle doit toujours mener le clinicien à penser à une éventuelle HTP en cause, notamment quand elle est associée à une hématémèse. Elle peut être asymptomatique de découverte fortuite au cours d'un examen clinique general. Comme, elle peut être retrouvée à l'exploration d'une douleur abdominale notamment de l'hypochondre gauche souvent en rapport avec un infarctus splénique. Elle peut prendre des dimensions considérables source de distension et d'inconfort, parfois dépassant l'ombilic et comblant la fosse iliaque. Habituellement responsable d'un hypersplénisme, elle ne ferait qu'aggraver les hématémèses et les épisodes d’épistaxis. Dans notre série une SMG était présent dans 100% des cas. Des résultats pareils ont été trouvés dans la littérature; plus de 80% pour Leger [2], 100% pour El Bouazzaoui [3] et 93% pour Dibi [1]. La c irculation veineuse collatérale est de topographie abdominale supérieure et thoracique basse et dans un sens de courant ascendant. C'est un signe principal d'une HTP, néanmoins il n'est pas constant. Elle était présente dans 15% pour Leger [2], dans 55% pour El Bouazzaoui [3] et dans 45% dans la série de Dibi [1]. Dans notre série, une CVC a été retrouvé dans 27% des cas. L'ascite est rare chez l'enfant atteint d'un cavernome portal et reste modérée et transitoire. Elle est présente dans 24%; 33% et 28% des cas selon les déférentes séries [1–3]. Dans notre série, l'ascite a été retrouvée dans 36% des cas rejoignant ainsi les données des autres séries. Les douleurs abdominales sont de siège variable, épigastrique, de l'hypochondre gauche. Ces douleurs abdominales peuvent être intenses et atypiques dans un tableau digestif non spécifique parfois dans un contexte fébrile en rapport avec une thrombose portale aigue ou en extension. L'hépatomégalie a été retrouvé dans notre série chez 27%et pour les autres séries une HMG a été notée respectivement dans 32%, 22%, 7% des cas. [1–3]. Dans notre série, le foie était normal dans 73% cas et hétérogène dans 27% cas. Un ictère peut être rencontré chez ces enfants atteints de cavernome porte, souvent suite à un épisode hémorragique mais exceptionnellement en rapport avec une biliopathie portale. Comme le montrent les études de la littérature il est rare chez l'enfant [9, 10]. L'ictère était absent dans notre série et dans les autres séries [2] a été observé dans 10%; et dans 11% des cas [3, 1]. La fièvre a été présente dans 11% des cas et 37% pour les séries rapportés dans la littérature [1, 2]. Nous avons noté dans notre série un seul cas fébrile. Le retard staturo-pondéral induit par une HTP extra-hépatique reste controversé. Dans notre série, on a objectivé le retard statural chez trois malades soit 27% des cas.

La biologie joue un rôle principal dans le diagnostic d'une HTP extra hépatique en permettant d'affirmer l'intégrité de la fonction hépatique et reflète clairement les conséquences de cette obstruction porte, en montrant le retentissement hématologique et hépatique. Elle permet aussi de détecter des anomalies héréditaires ou acquises des facteurs pro thrombotiques ou anticoagulants en faveur d'une thrombophilie. La fibroscopie œsogastroduodénale (FOGD) est devenue un examen inévitable devant toute hémorragie digestive. Elle est indiquée à visée diagnostique, pronostique et thérapeutique. Dans le cadre d'une HTP une FOGD permet de préciser la présence ou non de varices œsophagiennes, gastriques et ou d'une gastropathie hypertensive, ainsi que leur grade et le risque hémorragique, selon la classification modifiée de Stringer et Schwartz [11–13]. Dans la littérature, les varices œsophagiennes (VO) ont été visualisées au tiers inferieur de l’œsophage, pour les différentes séries dans 97%; 96%; 95% et 62% des cas [1, 3, 7, 14]. Les varices gastriques sont moins fréquentes, de siège principalement cardio-tuberositaire et sont considérées à haut risque hémorragique. Elles ont été retrouvées dans les séries avec des pourcentages de 25%; 23% et 12% des cas [1, 3, 7]. Dans notre série, les VO ont été trouvés dans 100% des cas; des varices gastriques dans 27% des cas; une gastropathie hypertensive dans 27% des cas et des varices duodénale dans un cas. L’échographie et doppler est C'est l'examen de première intention devant la découverte de signes cliniques digestifs de signes cliniques digestifs; et reste un moyen excellent de diagnostic ou voire pour une exploration abdominale de routine et reste un moyen excellent de diagnostic et de suivi d'un cavernome portal. Dans notre série elle a permis de poser le diagnostic de cavernome portal dans 100% des cas comme dans les autres séries. [1, 15]. La scannographie permet aussi de faire le diagnostic de thrombose portale. La TDM offre la possibilité de vues selon des plans coronal ou sagittal ce qui facilite la visualisation du thrombus sur tout le système porte. Cet examen a été pratiqué chez 3 de nos patients et il a a permet de confirmer le diagnostic de cavernome portal dans les 3cas avec description précise du trajet des diamètres des veines mésentériques et spléniques.

Le but du traitement est de supprimer le risque hémorragique sans pour autant ajouter des risques de complications propres aux différentes modalités thérapeutiques. Le traitement essentiel des hématémèses est constitué par les transfusions. Elles seront administrées en fonction de l'abondance du saignement et du taux d'hémoglobine et d'hématocrite et l'arrêt de saignement par tamponnement direct ou partraitement endoscopique par la ligature ou la sclérothérapie. Chez l′enfant, les mesures directes de l′effet des β-loquant a démontré une réduction de la pression splénique quand l′index cardiaque était réduit de 25%, cependant, il n′y avait pas d′études randomisées utilisant des β-loquants chez les enfants atteints d′HTP pour la prévention primaire des VO [16]. Le traitement chirurgical de l′HTP chez l′enfant a véritablement évolué au cours de cette dernière décennie. Cette chirurgie peut se faire de trois façons: Les Interventions non dérivatoires; Les dérivations porto-cave et qui visent à baisser la pression portale en contournant l'obstacle, pré hépatique dans notre contexte et le Pontage mésentérico-Rex ou reperfusion portale qui c'est une technique plus physiologique, et très prometteuse dans le traitement curatif des cavernomes portes. [17–20].

## Conclusion

Le cavernome portal est une cause majeure de l'hypertension portale en pédiatrie. Les manifestations cliniques sont habituellement en rapport avec une hypertension portale extra hépatique, les hémorragies digestives précoces et la splénomégalie étant les signes les plus fréquents dans la maladie, et rarement avec une compression biliaire. Par ailleurs, cette pathologie peut être responsable d'un retard de croissance chez l'enfant. Le diagnostic repose principalement sur l'imagerie. La performance de l’écho-doppler, l'angio-scanner et l'angio-IRM dans l'exploration du système porte a rendu les examens invasifs presque inutiles. Les modalités thérapeutiques médicales et notamment chirurgicales ont connu récemment un grand progrès ce qui a permis une prise en charge meilleure de ces malades. A la fin, nous concluons que le cavernome portal est une pathologie handicapante de l'enfant exigeant une prise en charge multidisciplinaire de haut niveau entre pédiatres, hépato-gastroentérologues, radiologues et chirurgiens infantiles, permettant de prendre une décision précoce et adaptée qui ne se réalisera qu’à travers une maîtrise des différents outils thérapeutiques.
